# Demographics, clinical characteristics, and recurrence rate of patients with primary spontaneous pneumothorax at a tertiary care center in Qatar

**DOI:** 10.5339/qmj.2022.56

**Published:** 2022-11-16

**Authors:** Adeel Ahmad Khan, Muhammad Zahid, Mousa Ahmad Alhiyari, Abdulrahman Ahmad Al-Andulmalek, Unwam Ekpuk Jumbo, Mohammad Naser Kloub, Muhammad Muslim, Muhammad Naeem, Zohaib Yousaf, Rashid Mazhar, Muhammad Sharif

**Affiliations:** ^1^Department of Medicine, Hamad Medical Corporation, Doha, Qatar Email & ORCID ID: adeel_1026@yahoo.com & https://orcid.org/0000-0003-1583-1539; ^2^Assistant Professor, Weill Cornell Medicine-Qatar; ^3^Thoracic Surgery Department, Hamad Medical Corporation, Doha, Qatar; ^4^Clinical Instructor, Weill Cornell Medicine-Qatar

**Keywords:** Pneumothorax, Primary Spontaneous Pneumothorax, Recurrence, Video-Assisted Thoracoscopic Surgery, VATS

## Abstract

Introduction: Primary spontaneous pneumothorax (PSP) is a common medical emergency. Its treatment includes simple observation, needle thoracentesis, pleural catheter, video-assisted thoracoscopy (VATS), and open surgery. We aimed to establish the demographic, clinical characteristics, and 12-month recurrence rate of patients with PSP in four hospitals of the Hamad Medical Corporation, Qatar.

Materials and methods: We conducted a retrospective analysis of patients >14 years old who were admitted with PSP from January 1, 2017 to December 31, 2019. The patients were followed up for 12 months for the recurrence of PSP.

Results: Out of 246 patients enrolled in this study, 223 (90.7%) were males and 23 (9.3%) were females. Their mean age was 29.1 ± 9.59 years and their mean body mass index (BMI) was 21.7 ± 4.22 kg/m^2^. Of these, 123 (51.2%) patients were smokers. Chest pain was the most common presenting complaint (82.5%). A total of 59 (23.98%) patients had a small pneumothorax, whereas 187 (76.01%) patients had a large pneumothorax.

Among the patients with small pneumothorax, 16 (28.33%) were managed through observation alone, 2 (3.33%) required needle aspiration, 15 (25%) required pleural catheter insertion, and 26 (44.06%) underwent surgical intervention for management. Out of 187 patients with large pneumothorax, 16 (8.6%) were managed through observation, 7 (3.76%) required needle aspiration, 73 (38.1%) required pleural catheter insertion, and 91 (48.6%) underwent surgery.

During the 12-month follow-up, 58 patients were lost to follow-up and 5 patients (5/188; 2.66%) presented with a recurrence of PSP. Out of 108 patients who underwent VATS, 2 (1.85%) had a recurrence of PSP.

Conclusion: PSP is more common in males than in females, with a tendency for younger age onset. The recurrence rate of PSP in our study was 2.66%. Furthermore, the recurrence rate in our patients who underwent VATS was 1.85%. Prospective studies are warranted to compare the success rates of different treatment interventions.

## Introduction

Pneumothorax refers to the presence of air in the pleural cavity that leads to a variable degree of lung collapse. Pneumothorax has a significant burden on health systems across the world. The reported incidence of pneumothorax is 18–28/100 000 cases per annum for males and 1.2–6/100,000 for females.^1^ It occurs more frequently in men than in women, possibly due to the differences in the associated risk factors, such as smoking and other lifestyle factors.^2^ The true incidence of primary spontaneous pneumothorax (PSP) in the expanding modern health system of Qatar remains unknown.

PSP is a common medical emergency, mainly affecting young adults who are tall and have a low BMI. It is called PSP, because, at the time of diagnosis, there is no apparent cause, including a trauma/iatrogenic or underlying lung disease. Secondary spontaneous pneumothorax (SSP) almost always occurs in older subjects with an underlying lung pathology or as a result of trauma. Due to the underlying lung pathology, SSP is more difficult to treat.^3^

The underlying etiology or risk factors that predispose PSP development include smoking, environmental factors, and genetic predisposition, which commonly manifests as apicomedial superficial bullae in the upper lobes. The demonstration of these apical pleural blebs/bullae on computed tomography (CT) or during thoracoscopic examination is approximately 90% in PSP.^4,5^ Smoking is the most important modifiable risk factor for the development of PSP. Heavy smokers are therefore at a greater risk than light smokers. A Swedish study reported a 12% higher risk of PSP in smokers than in nonsmokers.^6^ The likely pathophysiological mechanism is smoking-induced inflammation. In addition, the reoccurrence of PSP is more common in smokers.^7^

The most common symptoms include chest pain and shortness of breath. However, patients with small PSP ( < 2 cm at the level of hilum on chest X-ray (CXR) may have minimal or no symptoms. The clinical signs are variable and may include tachycardia, tachypnea, hypoxemia, and reduced breath sounds. Shifted trachea, which is a sign of large or tension pneumothorax, is rare in PSP.^3^

**Figure 1.**
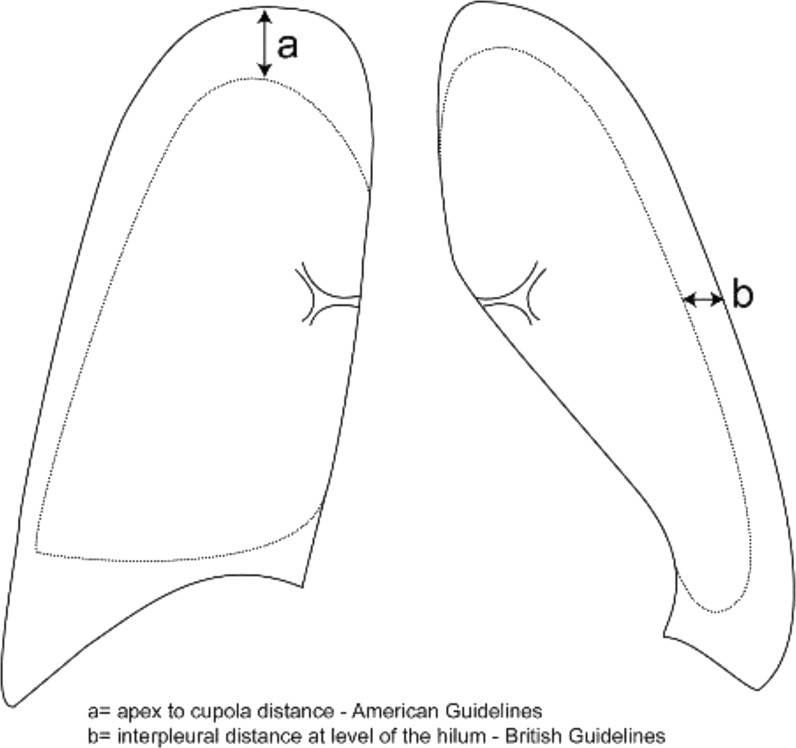


Diagnosis is confirmed through different imaging modalities, including CXR, thorax CT scan, and ultrasonography.^8^ The assessment of the severity of PSP is variable among different leading professional medical societies. For instance, the British Thoracic Society defines a “large” pneumothorax as one with a visible rim of pneumothorax >2 cm at the level of hila, between the chest wall and lung margin (>50% of the hemithorax volume involved) (Figure 1).^8^ The American College of Chest Physicians, on the other hand, uses 3 cm as the cutoff point.^9^

Different treatment modalities are available, and practices vary among clinicians, institutions, and even according to professional societies’ guidelines. The prescribed treatment may include simple observation with supplemental oxygen, large-bore needle thoracentesis, intercostal drain (ICD) placement, or video-assisted thoracoscopy (VATS).^8,9^

Importantly, Qatar has a population of 2.9 million with expatriates comprising 88% of the total population. Males dominate the population in terms of numbers (74.9%) due to the presence of several expatriate workers, which increases the cumulative population at risk of developing PSP in Qatar.^10^

## Research Design And Methods

### Study design

We conducted a cross-sectional, retrospective analysis of patients who were admitted to one of the four hospitals of Hamad Medical Corporation, Qatar, and were diagnosed with PSP from January 2017 to December 2019.

The primary outcome of the study was the recurrence of pneumothorax within 12 months after the first episode of PSP. The secondary outcomes included any association between age and recurrence, BMI and recurrence, characteristics of patients with recurrence, and management strategies in patients with recurrence.

### Inclusion criteria

All patients of age >14 years who were diagnosed with PSP and admitted to one of the four hospitals of Hamad Medical Corporation, Qatar, between January 2017 to December 2019 were included in the study.

### Exclusion criteria

Patients aged < 14 years and those with SSP were excluded from the study.

### Data collection

Data of patients who were diagnosed with PSP between 2017 and 2019 were abstracted from the electronic records of the Hamad Medical Corporation patient data repository. Data collected included demographics such as age, gender, ethnicity, body mass index (BMI), and comorbid conditions. Data related to patients’ pneumothorax included the size of pneumothorax on CXR, the types of interventions performed, and the recurrence of pneumothorax within 12 months of intervention.

### Statistical analyses

Descriptive statistics were applied to describe the study cohort's demographic parameters, with continuous variables presented as the means ±  standard deviation or median (interquartile range), as deemed appropriate. In contrast, categorical variables were presented as numbers (percentages). All data were analyzed using the Jamovi version 1.2 (created in 2020, Sydney, Australia).

The ethical approval for the study was provided by the Medical Research Center (MRC; ID no. MRC 01-20-600)

## Results

Out of 246 patients, 223 (90.7%) were males and 23 (9.3%) were females. A total of 90 (36.6%) patients were Qatari and 156 (63.4%) were non-Qatari. Their mean age was 29.1 ± 9.59 years and their mean BMI was 21.7 ± 4.22 kg/m^2^. A total of 123 (51.2%) patients were smokers, 103 (42.9%) were nonsmokers, and 14 (5.8%) patients were exsmokers. In terms of comorbidities, only 6 (2.4%) patients had hypertension and 1 (0.4%) had diabetes mellitus (Table 1).

Chest pain was the most common presenting complaint (82.5%), followed by shortness of breath (55.5%) and cough (17.1%). A total of 59 (23.98) patients had small ( < 2 cm) pneumothorax on the CXR, whereas 187 (76.01%) patients had a large pneumothorax (>2 cm) on the CXR. A total of 35 (14.2%) patients had blebs, while 64 (26.01%) had bullae on the CT thorax (Table 1).

Among the patients with small pneumothorax, 16 (28.33%) were managed successfully with observation alone, while 2 (3.33%) were treated with needle aspiration, 15 (25%) were managed with a pleural catheter insertion, and 26 (44.06%) required surgical intervention for management (Table 2). On the other hand, out of 187 patients with large pneumothorax, 16 (8.6%) were managed with observation, 7 (3.76%) with needle aspiration, 73 (38.1%) with pleural catheter insertion, and 91 (48.6%) with surgical intervention (Table 3). The types of surgical interventions performed are depicted in Table 4. The mean length of stay in patients with the nonsurgical intervention was 4.62 ± 5.85 days, while in patients with surgical intervention, it was 8.64 ± 6.75 days (*p* < 0.0001, CI 2.43–5.61).

Out of 246 patients, 188 completed the 12-month follow-up. A total of 5 patients had a recurrence of pneumothorax (2.66%). In 4 of these cases, the recurrence was ipsilateral, while the fifth patient developed bilateral pneumothorax during recurrence. Of the 5, 2 patients were males and 3 were females. Their mean age was 23.8 ± 9.311 years, while their mean BMI was 22.382 ± 3.99 kg/m^2^. Two patients had a history of smoking, while the other 3 were nonsmokers. A statistically significant association was observed between lower age and recurrence (*p* < 0.001). No such association was observed between BMI and the risk of recurrence. Out of 5 patients with recurrence, 1 had an initial small pneumothorax, while the remaining 4 had large initial pneumothoraces. In 2 patients, the initial large pneumothorax episodes were managed through observation alone. The recurrent episodes were successfully managed with pleural catheter insertion. One patient (initial episode of large left-sided pneumothorax managed with ICD) had a recurrence involving both lungs. This bilateral pneumothorax was managed in this patient successfully, with bilateral pleural catheter insertion (Table 5).

Two patients had a recurrence of pneumothorax after the VAT. One of them had a history of right-sided pneumothorax in 2013, which required VATS performed outside Qatar (Patient #1 in Table 5). In 2017, this patient again developed a right-sided small pneumothorax, which was managed with VATS (apical lung tissue resection, pleurectomy, and pleural abrasion). One year later, she presented again with a right-sided small pneumothorax, which was successfully managed with observation alone. The second patient had an initial episode of right-sided large pneumothorax and was treated with VATS (bullectomy). He developed ipsilateral recurrence, which was again large (>2 cm). However, he refused any intervention and was lost to follow-up (Table 4).

## Discussion

PSP is more common in men than women, with literature reporting a male-to-female ratio of up to 3.3:1.^11^ Furthermore, females with PSP tend to be older as compared to males.^12^ Our cohort of PSP patients included 90% males, with a female-to-male ratio of 1:9, and the mean age of females was higher than that of males. The sex ratio of the total population in Qatar is 3.150 (3,150 males per 1,000 females), which is higher than the global sex ratio.^13^ This can be attributed to the peculiar demographic distribution of the population in Qatar, with the majority being young male adult workers from South East Asia. Pneumothorax is more common among males than females because smoking is more prevalent among males, and males start smoking earlier than females.^6^ As males have smaller airways than females, airway obstruction, which is characteristic of pneumothorax, may manifest earlier in them.^14^ Smoking increases the risk of developing PSP by 22-fold in males.^6^ In a Qatar-based study conducted by AlMulla *et al.*,^15^ the prevalence of current tobacco use among adult Qataris and non-Qataris was 25.2% and 21.5%, respectively. In another study by Vishal,^16^ tobacco use was significantly higher among males (10%) than among females (1.1%). These results may explain the high prevalence of PSP among males in Qatar. Furthermore, in our cohort, 67.8% of Qatari patients were active smokers, while 39.7% of expatriates were active smokers at the time of diagnosis of PSP, with the difference in two proportions being statistically significant (*p* < 0.001).

In a study of 153 patients with spontaneous pneumothorax, 9 patients were initially managed with aspiration alone, 6 with aspiration followed by pleural catheter insertion, 14 with observation, and 124 with pleural catheter insertion.^17^ Paoloni et al.^18^ reported that 32% patient of PSP were managed with observation, 20% with aspiration, and 48% with pleural catheter/ICD insertion.^18^ In our study, 13% of patients were managed with observation alone, 3.65% with needle aspiration alone, 35.8% with pleural catheter insertion, and 47.6% through definitive management.

Past studies have reported different rates of recurrence in patients with PSP. A meta-analysis of 29 studies, conducted by Walker et al.^19^ concluded that the recurrence rate in PSP was 32% with a mean duration of follow-up of 3–96 months. A study from Kuwait showed a recurrence rate of 34.6%, with approximately 62.5% of these recurrences occurring within the first year.^20^ Our study found that the rate of recurrence was 2.66% in the first year following PSP. An association between BMI and the risk of PSP recurrence remains controversial. A retrospective cohort study conducted by Tan et al.^21^ reported that the association between low BMI and the risk of recurrence was statistically significant. Olesen et al.^22^ also reported a low body weight ( < 60 kg) as an independent risk factor associated with recurrence. Noh et al.^23^ reported no such association between BMI and the risk of recurrence. In our study, no statistically significant association was recorded between BMI and the risk of recurrence.

Several studies have reported younger age as a risk factor for the recurrence of PSP. Huang et al.^24^ reported a more significant proportion of patients having a recurrence of PSP in patients belonging to the younger age group. Noh et al.^23^ also reported a higher recurrence rate in younger patients with PSP who underwent wedge resection. In our study too, lower age was associated with an increased risk of recurrence. However, the sample size of patients with recurrence in our study was too small to detect any such difference.

After the first episode, indications for surgical management of PSP included people belonging to high-risk professions (such as high-altitude dwellers, scuba divers, pilots, climbers, and skiers) or those living long distances from the nearest hospital. VATS is the surgical procedure of choice by most thoracic surgeons.^25^ In our cohort of patients, VATS was the most frequently performed surgical intervention (108/117, 92.3%). At our institution, VATS for PSP invariably involved bullectomy with parietal pleurectomy of the upper hemithorax and scratch pad abrasion of the lower half of the hemithorax. VATS has been reported to be the most common surgical procedure by other studies as well.^11^ VATS as a treatment modality after the first episode of PSP has been reported to be safe, with low morbidity, reduced length of stay in the hospital, and better patient satisfaction by several studies.^7^

Definitive surgical management of PSP with VATS has been reported to have a lower risk of pneumothorax recurrence. A prospective study of 1415 patients reported the incidence of pneumothorax recurrence to be 1.9% after VATS.^28^ Tan et al.^21^ also reported the risk of recurrence to be significantly lower in patients who had preventive surgical intervention (i.e., VATS) when compared with those who were managed with nonpreventive interventions (such as observation, needle observation, and chest drain insertion). Brysch et Al.^29^ reported the recurrence rate of PSP following VATS as 1.3%. Out of 108 patients who underwent VATS in our study, the recurrence rate was 1.85% (2 patients). The mean age in Qatari patients with PSP in our cohort was 25.5 ± 9.5 years. As per 2019 data, nearly 45.8% of the Qatari population belongs to this age group (15–34 years) and therefore are at risk of developing PSP.^30^ A low recurrence rate of PSP in our cohort is multifactorial, with the early definitive intervention being one of the factors.

One major limitation of our study is the loss of follow-up of 58 (23.6%) patients, 38 (65.6%) of which were expatriates. This could be due to the expatriate workers leaving the country. However, it might affect the true recurrence rate of PSP in our study.

## Conclusion

PSP is more common in smokers, males, and in young adults. The recurrence rate of PSP in our study was 2.66%, which is significantly lower than the one reported in the literature. Furthermore, the recurrence rate in patients who underwent VATS was 1.85%. There is a possible association between lower age and an increased risk of recurrence of PSP. However, the sample size of patients with recurrence is very small and warrants studies with larger sample sizes.

## Disclosures

### Ethics declaration

This work is original, has not been, and is not under consideration for publication in any other Journal. All authors have reviewed and approved the final version of the manuscript. The study was approved by the MRC Qatar.

### Data sharing

Available from the corresponding author upon reasonable request.

### Approval of the research protocol

The research protocol for the study was approved by the MRC at Hamad Medical Corporation with protocol ID number (MRC-01-20-836)

### Conflict of interest

None of the authors have any conflict of interest to disclose.

## Figures and Tables

**Table 1:** Baseline demographic data of the study patients

N	246

Male	223 (90.7%)

Female	23 (9.3%)

Overall Age (years)	29.1+/-9.59

Mean age in females (years)	36.6+/-12.1

Mean age in males (years)	28.3+/-9

Mean age in Qatari patients (years)	25.5+/-9.5

Overall mean Body mass index (BMI-kg/m^2^)	21.7+/-4.22

mean Body mass index in females (BMI-kg/m^2^)	21.7+/-2.84

mean Body mass index in males (BMI-kg/m^2^)	21.7+/-4.34

mean Body mass index Qatari patients (BMI-kg/m^2^)	21.1+/-4.48

Nationality	

Qatari	90 (36.6%)

Non-Qatari	156 (63.4%)

• Indian	33 (13.4%)

• Filipino	16 (6.5%)

• Bangladeshi	13 (5.3%)

• Syrian	10 (4.1%)

• Egyptian	9 (3.7%)

• Pakistani	5 (2%)

• Jordanian	3 (1.2%)

• Nepalese	3 (1.2%)

• Srilankan	2 (0.8%)

• Yemini	2 (0.8%)

• American	1 (0.4%)

• British	1 (0.4%)

• Kuwaiti	1 (0.4%)

• Moroccan	1 (0.4%)

• Palestinian	1 (0.4%)

• Somali	1 (0.4%)

• Ugandan	1 (0.4%)

• Unknown/Other nationalities	53 (21.5%)

Smoker	123 (50%)

Non-smoker	103 (41.8%)

Ex-smoker	14 (5.7%)

Smoking status undetermined	6 (2.4)

Comorbidities	

Diabetes mellitus	1 (0.4%)

Hypertension	6 (2.4%)

Coronary artery disease	0

Asthma	0

Interstitial lung disease	0

Presenting complaint	

Cough	42 (17.1%)

Shortness of breath	137 (55.5%)

Chest pain	203 (82.5%)

Number of patients with blebs on CT chest	35 (14.2%)

Number of patients with blebs on CT chest	64 (26.01%)


**Table 2:** Interventions in patients with pneumothorax < 2 cm on chest X-ray

Type of intervention	Number of patients (%) n = 59

Observation alone	16 (28.33%)

Needle aspiration	2 (3.33%)

Pleural catheter	15 (25%)

Surgical treatment (VATS, open surgery, robotic surgery)	26 (44.06%)


VATS: Video-assisted thoracoscopic surgery

**Table 3:** Interventions in patients with pneumothorax >2 cm on chest X-ray

Types of interventions	Number of patients (%) n = 187

Pleural catheter	73 (38.1%)

Observation alone	16 (8.6%)

Needle aspiration	7 (3.76%)

Surgical management (VATS, robotics surgery, open surgery)	91 (48.6%)


VATS: Video-assisted thoracoscopic surgery

**Table 4:** Types of surgical interventions undertaken for pneumothorax

Surgical intervention	Number

VATS	108

• Bullectomy + pleural abrasion	19

• Bullectomy + upper Pleurectomy and lower pleural abrasion	82

• Bullectomy alone	4

• Surgery + Talc insufflation	3

Open surgery	4

• Bullectomy + Pleural Abrasion	2

• Bullectomy + upper Pleurectomy and lower pleural abrasion	2

Robotics	5

• Bullectomy + Pleural Abrasion	1

• Bullectomy + upper Pleurectomy and lower pleural abrasion	4

	


VATS: Video-assisted thoracoscopic surgery

**Table 5:** Characteristics of patients with a recurrence of pneumothorax

	Gender	Age	Smoker (Yes/No)	Side of PTX (initial episode)	Size of PTX on CXR (initial episode)	Intervention (initial episode)	Side of PTX (recurrent episode)	Size of PTX on CXR (recurrent episode)	Intervention (recurrent episode)

#Patient 1	Female	40	No	Right side	< 2 cm	VATS (apical lung tissue resection, pleurectomy, and pleural abrasions)	Right side	< 2 cm	Observation

Patient 2	Male	16	Yes	Left side	>2 cm	Observation	Left side	>2 cm	Pleural catheter

Patient 3	Male	21	No	Right side	>2 cm	VATS (Bullectomy)	Right side	>2 cm	The patient refused intervention (lost to follow-up)

Patient 4	Female	21	Yes	Left side	>2 cm	Observation	Left side	>2 cm	Pleural catheter

Patient 5	Female	21	No	Left side	>2 cm	Pleural catheter	Bilateral PTX	>2 cm	Pleural catheter


PTX: pneumothorax; CXR: Chest X-ray; VATS: Video-assisted thoracoscopic surgery
